# Automatic machine-learning based identification of jogging periods from accelerometer measurements of adolescents under field conditions

**DOI:** 10.1371/journal.pone.0184216

**Published:** 2017-09-07

**Authors:** Eftim Zdravevski, Biljana Risteska Stojkoska, Marie Standl, Holger Schulz

**Affiliations:** 1 Faculty of Computer Science and Engineering, Saints Cyril and Methodius University, Skopje, Macedonia; 2 Institute of Epidemiology I, Helmholtz Zentrum München, German Research Center for Environmental Health, Neuherberg, Germany; Weill Cornell Medical College in Qatar, QATAR

## Abstract

**Background:**

Assessment of health benefits associated with physical activity depend on the activity duration, intensity and frequency, therefore their correct identification is very valuable and important in epidemiological and clinical studies. The aims of this study are: to develop an algorithm for automatic identification of intended jogging periods; and to assess whether the identification performance is improved when using two accelerometers at the hip and ankle, compared to when using only one at either position.

**Methods:**

The study used diarized jogging periods and the corresponding accelerometer data from thirty-nine, 15-year-old adolescents, collected under field conditions, as part of the GINIplus study. The data was obtained from two accelerometers placed at the hip and ankle. Automated feature engineering technique was performed to extract features from the raw accelerometer readings and to select a subset of the most significant features. Four machine learning algorithms were used for classification: Logistic regression, Support Vector Machines, Random Forest and Extremely Randomized Trees. Classification was performed using only data from the hip accelerometer, using only data from ankle accelerometer and using data from both accelerometers.

**Results:**

The reported jogging periods were verified by visual inspection and used as golden standard. After the feature selection and tuning of the classification algorithms, all options provided a classification accuracy of at least 0.99, independent of the applied segmentation strategy with sliding windows of either 60s or 180s. The best matching ratio, i.e. the length of correctly identified jogging periods related to the total time including the missed ones, was up to 0.875. It could be additionally improved up to 0.967 by application of post-classification rules, which considered the duration of breaks and jogging periods. There was no obvious benefit of using two accelerometers, rather almost the same performance could be achieved from either accelerometer position.

**Conclusions:**

Machine learning techniques can be used for automatic activity recognition, as they provide very accurate activity recognition, significantly more accurate than when keeping a diary. Identification of jogging periods in adolescents can be performed using only one accelerometer. Performance-wise there is no significant benefit from using accelerometers on both locations.

## Introduction

Physical activity (PA) is a major life style factor associated with beneficial health effects across the life span [1–3]. PA reduces the risk and progression of chronic diseases common in the developed world by improving functional abilities, cardiorespiratory fitness and metabolic health in patients with frequent diseases, such as cardiovascular, lung, and neurodegenerative diseases, but also in the healthy population [4–13]. Since intensity, duration, and frequency of PA have a great impact on PA associated health benefits [1–3], assessment of PA has become a standard parameter in many epidemiological and clinical studies. Historically, standardized questionnaires, such as PASE (Physical Activity Scale for the Elderly) [14] have been applied for PA assessment. In recent years, sensors monitoring ambulation, such as pedometers or accelerometers, are increasingly used for assessment of PA [15–17]. This is related to the fact that self-reporting of PA tends to overestimate the time being physically active, particularly in high intensity levels [18–20]. Accelerometers provide an objective measurement of acceleration throughout the day, thus allowing more precise estimates of PA levels and patterns, e.g. determination of the daily periods spent in moderate-to-vigorous PA in bouts of at least 10 minutes.

Current methods for PA evaluation applied in epidemiological studies [2, 21, 22] do not provide information about the type of activity nor give an idea about the sport performed by the participant. Therefore, this information must be reported separately by the participant in an activity diary [21, 22] and may lack accuracy with respect to time spent performing particular activity. Moreover, requiring participants to keep an activity diary may reduce their compliance to take part in the study.

Meanwhile, in studies related to ambient assisted living, recognition of activities of daily living based on accelerometer recordings has become very popular [23–33]. Generally, such approaches first segment the time series data with sliding windows, then apply signal processing and statistical methods for feature extraction from the raw accelerometer measurements and then train machine learning algorithms for classification of different activities. The main limitation of these studies is that they are based on measurements in controlled environments with small number of participants and very limited duration of the experiments [24–33]. According to [23], systematic and automated feature engineering and selection which initially considers variety of features is superior to approaches [30–33] based on hand-tailored features for a particular set of sensor types, body placement or study goal.

The study presented in this article aims to develop a new algorithm based on machine learning techniques for detection of intended jogging periods, using conventional accelerometer data recorded under field conditions. To the best of our knowledge, this is the first study that uses data collected under field conditions for building models for activity recognition. The data used in the study is comprised of measurements from two accelerometers placed at the hip and ankle of adolescents and the corresponding activity diary. Additionally, this study aims to determine whether recordings of two accelerometers, i.e. monitoring of body movement at two different positions, is required or whether one position is sufficient for accurate identification of jogging periods.

Jogging provides regular rhythmically movements and thus is supposed to be easily identifiable by an algorithm. Moreover, it is among the most popular leisure-time sporting activities in the population with an increasing trend in the past years [34, 35] and is linked with significant health benefits [35–38]. A recent meta-analysis of interventional jogging studies conducted in healthy 18-65 year old inactive adults [39] showed that after 1 year of habitual running, many benefits were observed, like reductions in body weight and fat, resting heart rate, increased high density lipoprotein (HDL) cholesterol and an improved maximal oxygen uptake. These biomedical indices correlated directly with the running intensity. Regular runners have a substantially reduced risk for premature mortality and their life expectancy is increased by about 3 years [35]. Running is protective against common chronic diseases, e.g. the risk of cardiovascular mortality is reduced 45-70% compared to non-runners [35]. Further benefits are reported for metabolic fitness, metabolic processes, the muscoskeletal, the cardiovascular, the endocrinological, and the neurological system [35–38]. Current recommendations include jogging periods at a relative modest pace for 15-30 minutes on 3 to 7 days a week [37, 40]. Thus, identification of jogging periods in accelerometer recordings from epidemiological or clinical studies is of great scientific interest.

## Methods and analysis

The standard machine learning approach [41] for activity recognition is applied in this study. First, the raw accelerometer readings are segmented with the sliding windows technique, and from each window time and frequency domain features are extracted. Using feature selection algorithms, the number of features is reduced, aiming to get more robust classification models and to reduce the machine learning algorithms training time [42]. Then, several machine learning algorithms are applied to generate classification models for jogging periods recognition.

### Ethics statement

The German Infant Nutrition Intervention Programme PLUS environmental and genetic influences on allergy development (GINIPlus) study was approved by the local Ethics Committees, the Bavarian General Medical Council (Bayerische Landesärztekammer, Munich, Germany) for the study place Munich and the Medical Council of North-Rhine-Westphalia (Ärztekammer Nordrhein, Düsseldorf, Germany) for Wesel. The approval of the Ethics Committees includes the written consent procedure. Written informed consent was obtained from the parents or the legal guardian of all participating adolescents.

### Instruments

To assess the PA of the study sample, two ActiGraph GT3X (Pensacola, FL) accelerometers were used. The ActiGraph GT3X contains a triaxial accelerometer for assessing accelerations in the vertical (Axis 1), horizontal (i.e. antero-posterior or Axis 2) and medio-lateral (Axis 3) axes [43]. ActiGraph (Pensacola, Florida, FL) is the most widely used accelerometer for assessing physical activity, therefore ActiGraph accelerometers were chosen for reasons of application and comparability to other studies. ActiLife software was used for initialization of accelerometers (version 5.5.5, firmware 4.4.0).

All feature extraction and machine learning algorithms were implemented in Python 3. We have used SciPy [44] and NumPy [45] libraries for the statistical calculations, and the scikit-learn library [46] for the classification algorithms. The algorithms were executed on a 2.3GHz quad-core Intel Core i7 processor (with Turbo Boost up to 3.3GHz) with 6MB L3 cache and 16 GB RAM.

### Participants

Measurements of physical activity (PA) by accelerometry were embedded in the 15-year follow-up of the GINIPlus. The cohort and recruitment of the participants has been described in [22, 47]. Briefly, 5991 newborns were enrolled in the cities of Munich and Wesel in Germany, of which 53% participated in the 15-year follow up (3198 adolescents). Of these, 1890 (59%) consented to accelerometry and 1054 provided data of acceptable quality for inclusion.

### Data management

The participants were instructed to wear the two accelerometers on their hip and ankle during the course of one week. The week started on the first school day after they received the accelerometers. During the day, one accelerometer was attached to an elastic belt at the hip on the side of the dominant hand. The other accelerometer was placed at the dominant ankle. Participants were required to keep a diary of all sport activities including intended jogging activities in a provided sheet, in which they entered the date, start time and end time of the intended jogging period, accurate up to a minute [21, 22]. Out of 1054 participants 626 reported sporting activities of whom 95 (28 male and 67 female) reported periods of jogging [21]. 39 participants were randomly selected and divided into three subsets: 14 for training, 13 for validation and 12 for testing of the machine learning algorithms. The diary record was verified as follows: for the reported jogging period plus/minus x additional minutes raw signals from hip and ankle accelerometer were graphically displayed and if applicable corrected according to the apparent start and end or short brakes of jogging.

The accelerometers sampling rate was set to 30 Hz and measured acceleration was stored at 1 Hz after conversion into proprietary ‘activity count units’ summed over a second-by-second time interval. Data filtering was set to default (‘normal’) recommended by ActiGraph. Activity counts of all three axes (vertical, horizontal and medio-lateral), the inclinometer signal, and the number of steps were measured. For each participant there was a file with the following 8 columns: Date, Time, *Axis 1*, *Axis 2*, *Axis 3*, *Steps*, *Inclinometer* and *Vector Magnitude*. Consequently, the last 6 columns represent *6 time series*. Because there are two accelerometers, placed at the hip and at the ankle of the participants, there are 12 time series available in total. Each row in these files represent the accelerometer readings at a particular moment (with precision of 1 second, corresponding to the 1 Hz storage rate).

**Fig 1:**
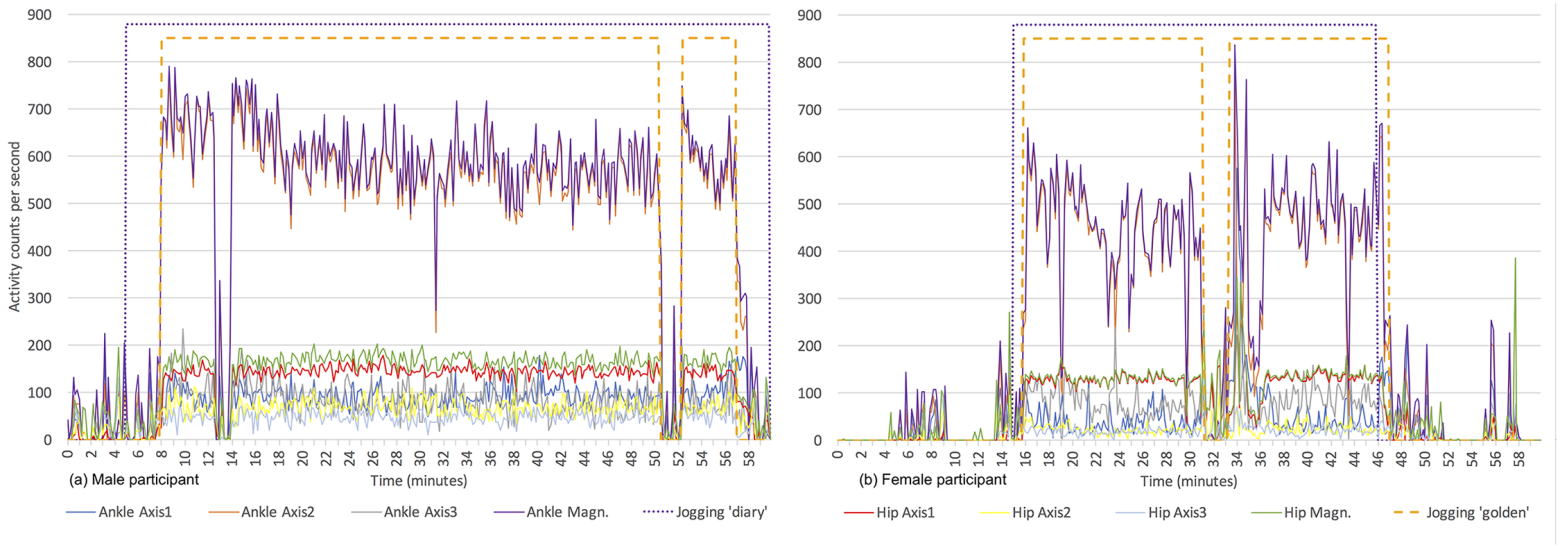
Exemplary raw accelerometer readings for one hour during which two participants, (a) male and (b) female, had a jogging activity. The inclinometer and number of steps time series are not shown for clarity because they are in a different unit with much smaller values. Jogging ‘diary’ relates to the reported jogging period by the user. Jogging ‘golden’ is the jogging period per the ‘golden standard’ labels.

For the selected participants, any errors in the self-reported labels or incorrect alignment with the recorded data (e.g. unsynchronized time on the user’s clock and accelerometer clock) have been fixed. Hereafter, the corrected logs are referred to as ‘golden standard’ labels, whereas the original diary entries as ‘diary’ labels, respectively. The difference between them is shown in Fig 1 together with the measurements from both accelerometers for a male (left) and a female (right) participant.

### Sample selection

From the whole set of participants with acceptable data quality, 39 participants were randomly selected and divided into three subsets: 14 for training, 13 for validation and 12 for testing of the machine learning algorithms (see Table 1). The gender distribution in the training and validation set was balanced, whereas in the test set it was completely random. The activity duration provided in Table 1 is based on the golden standard labels.

**Table 1:** Distribution of participants and Jogging Periods (JP) duration in minutes per dataset.

	Gender			JP Duration (minutes)				
Dataset	Male	Female	Days	Number of JP	Sum	Mean	Min	Max
Train	7	7	22	40	806	20.1	2	90
Validation	7	6	19	31	648	22.0	3	63
Test	4	8	14	39	380	9.8	1	46

Days is the total number of days on which participants reported a jogging period.

Using data from 39 participants is more than the number of participants used in other studies related to activity recognition: [30] (19 participants), [31] (10 participants), [32] (10 participants) and [33] (30 participants). The total recorded activity in these studies is very short (i.e. usually only several hours). In contrast, in our study we processed data from 55 days on which there was at least one intended jogging period (see Table 1).

### Data preparation

An overview of the data preparation steps commonly used in activity recognition systems is provided in [41]. At the beginning, data needs to be segmented with the sliding windows technique, where raw accelerometer measurements are divided into windows (i.e. segments) that contain measurements that happened in that particular time window. The adjacent windows can contain distinct data, or they can overlap, meaning that some measurements can belong to two consecutive windows. Generally, lower sensor frequencies or more complex activities entail longer windows. For recognition of jogging activities, a window of couple of seconds would suffice [41] if the sensor frequency is high (e.g. greater than 30 Hz). In our case, the window length needs to be much longer than couple of seconds for the following reasons: (i) the goal is not to recognize isolated short bursts of running (e.g. running to catch the bus), rather longer periods of intended jogging activities; (ii) short pauses within one longer jogging period (e.g. waiting on a traffic light before crossing a street) are allowed in this problem domain (iii) the data frequency from the available accelerometers is 1 Hz; (iv) the available labels from the golden standard and diary labels were accurate up to a minute, thus requiring the window length to be at least one minute.

**Fig 2:**
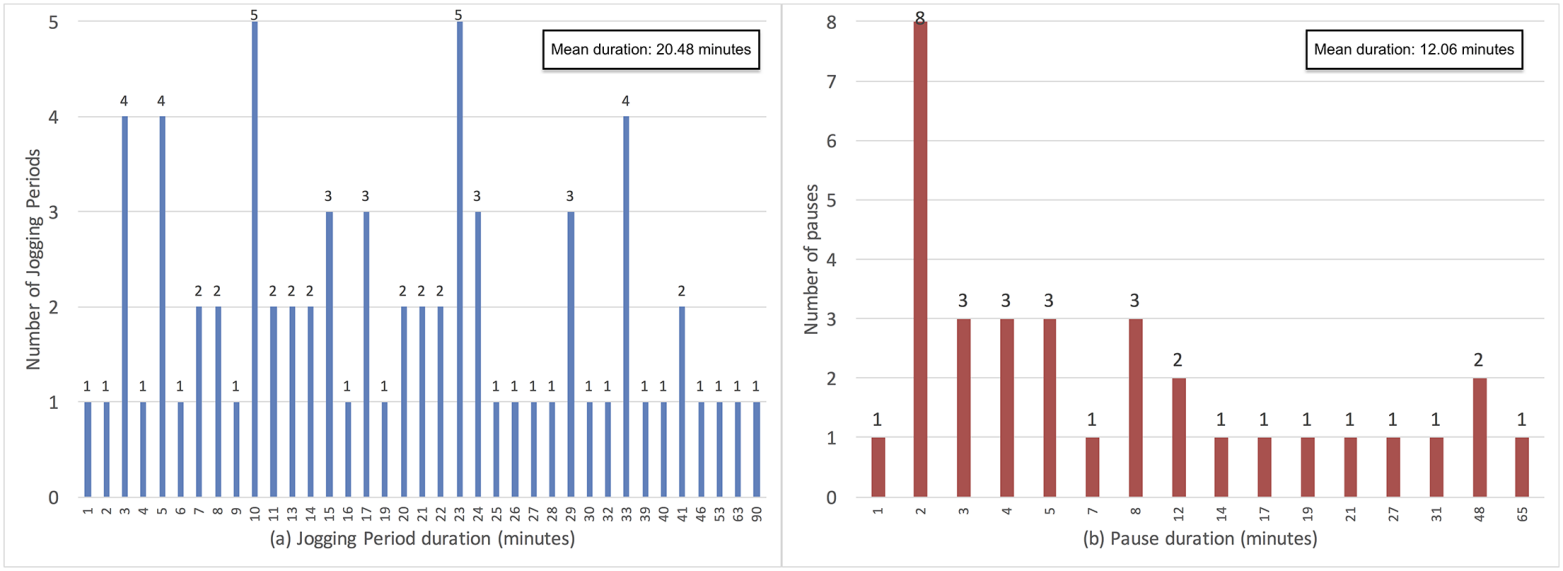
Distribution of the duration (in minutes) of jogging periods (a) and pauses between jogging periods (b) in the training and validation datasets based on the ‘golden standard’ labels before application of post-classification rules.

Therefore, we have decided to use two sliding window segmentation strategies: 60s windows without overlap, and 180s windows with 120s overlap, i.e. 60s shift between consecutive windows. Both window lengths are greater than the typical short pauses within jogging periods (see Fig 2(b)), and yet significantly smaller than the usual duration of jogging periods (see Fig 2(a)). More details about the obtained number of instances (i.e. epochs or episodes) with the two segmentation strategies for each dataset are provided in Table 2.

**Table 2:** Number of instances per dataset and segmentation strategy.

	60s windows without overlap			180s windows with 120s overlap		
Dataset	Jogging	Non-jogging	JR	Jogging	Non-jogging	JR
Train	829	31421	0.0257	745	31320	0.0232
Validation	707	27148	0.0254	645	27060	0.0233
Test	395	23437	0.0166	320	22966	0.0137

Jogging and Non-jogging is the number of instances (i.e. epochs or episodes) in the dataset and JR is the Jogging Ratio (i.e. Jogging/(Jogging+NonJogging)).

When the task is defined as a binary classification problem (i.e. jogging vs non-jogging), there is high class imbalance because the jogging periods are considerably shorter than the whole period of recorded activities (see Table 2), which degrades classifier performance and needs to be addressed [48]. Aiming to mitigate this, we considered only days on which participants reported a jogging activity.

### Classification algorithms

In this section we describe the classification algorithms used for feature ranking, wrapper feature selection and building classification models. Different classification models were compared in terms of accuracy, which represents the proportion of correctly recognized instances (both true epochs of jogging and true epochs of non-jogging) among the total number of instances (i.e. epochs).

(i) *Logistic Regression* [49] has many advantages, as its simplicity, portability, easy interpretation of classification models and importance of features, parallelization ability and speed. It is an essential part of the wrapper feature selection, described later in the manuscript.

(ii) *Random Forest* (RF) [50] is an efficient algorithm that generates multiple decision trees by randomly sampling instances from the dataset and randomly selecting *m* features in each sample from all *M* features. The tree branching is performed by finding the best split from the features on each node. In the process of classification, each tree votes for the class and the majority class is chosen. The default value of *m* is $\sqrt{M}$ in the scikit-learn library [46].

(iii) *Extremely Randomized Trees* (ERT) algorithm [51] is similar to RF, as it also generates an ensemble of trees. However, unlike RF, ERT chooses the split of the features randomly. This increases the training speed because the number of calculations per node is decreased. Both algorithms provide excellent classification performance and can train classification models on very large datasets very fast.

Both ERT and RF provide feature importance estimates, which is a property used for feature ranking and discarding of low-importance features in the feature selection phase. Our experiments showed that the importances generated by both algorithms are very consistent regardless of the number of features and trees, therefore we have chosen ERT because of its better speed. Using smaller number of trees is obviously faster, but depending on the feature set, the predictive performance sometimes is slightly worse.

For both algorithms the number of features in each feature subset, *m*, along with the number of trees in the ensemble, could potentially influence the classification performance, the required time for building models and the feature importance estimates. To investigate the effect of these parameters, we have repeated the experiments using the dataset obtained with segmentation strategy of 60s windows without overlap. For the *m* we have tested 5 equally distanced values in the following range: $\left[\frac{\sqrt{M}}{2},2\sqrt{M}\right]$. For the number of trees in the ensembles we have tested the following values: 100, 500, 1000 and 2000. Thus, a total of 20 combinations were evaluated on the unfiltered feature set for both ERT and RF, and this was repeated 5 times. The impact of the *m* value on the classification performance, duration and feature importance estimates was negligible regardless of the number of trees in the ensemble. The number of trees had a more noticeable impact on the training time, i.e. the standard deviation of the accuracy of different repetitions lowered as the number of trees increased. There was a small difference between 500 and 1000 trees, whereas between 1000 and 2000 the difference diminished. When repeating the same analysis on the datasets after the feature selection, the number of trees did not have any impact on accuracy while impacting only the training time. However, because 1000 trees provided optimal results for the unfiltered feature sets, we decided to always use 1000 trees for the ERT and RF classifiers.

(iv) *Support Vector Machines* (SVM) classifier [52] with Gaussian kernel is much slower algorithm as the dimensionality of data increases, but is very powerful, especially after parameter tuning [53]. C and *γ* are the parameters for a nonlinear SVM with a Gaussian radial basis function kernel. C is the cost of classification and a large C provides low bias and high variance. The Gaussian kernel, which aids in handling non-linear classification, has an additional parameter, *γ*, which is the free parameter of the Gaussian radial basis function. A small value of *γ* results in low bias and high variance. Parameter optimization is performed by evaluating various combinations of SVM parameters (in this study we evaluated C values ranging in 0.1, 1 and 10, and *γ* ranging in 0.01, 0.001, 0.0001 and 0.00001) using the training and validation datasets for a particular feature set [53]. Whenever we used SVMs, the datasets were normalized, so the training dataset will have mean and standard deviation of 0 and 1, respectively.

### Types of extracted features

The feature extraction consists of several steps, involving the 12 originally recorded time series and some newly generated time series from which a variety of features are generated.

Let *n* denote the number of measurements within one window (*n* ∈ 60,180 in this study). Let *x*_*i*_, where 0 ≤ *i* < *n*, represent the value of *j*-th time series within a particular sliding window. From all original time series, several types of features are extracted, which have been proven to be effective predictors in recent competitions [54, 55] related to feature extraction from a variety of time series data.

#### Basic statistics

The following basic statistics have been successfully used as informative features in various studies [56–60]: minimum (*min* = min *x*_*i*_), maximum (*max* = max *x*_*i*_), range (*max* − *min*), arithmetic mean ($\bar{x}=\sum x_{i}/n$), harmonic mean ($n/\sum x_{i}^{-1}$), geometric mean ((∏*x*_*i*_)^1/*n*^), mode (the value of the current time series that occurs most often in the current sliding window), standard deviation ($\sigma =\sqrt{\frac{1}{n-1}\sum {\left(x_{i}-\bar{x}\right)}^{2}}$) and variance (i.e. unbiased sample variance, *σ*^2^).

Skewness (the third central moment) and kurtosis (the fourth central moment) could also be useful features, computed per the definition in Section 2.2.24.1 and Section 2.2.25.1 in [61], respectively.

Additionally, these features were calculated: signal-to-noise ratio ($snr=\bar{x}/\sigma$), energy ($energy=\sum_{i=0}^{n-1}x_{i}$), and energy per sample (*energy*/*n*). Consequently, in total 14 features related to basic statistics are generated from each sliding window segment of a time series.

#### Equal-width histogram

In [56] it was shown that histogram-related features can be informative and robust for activity recognition. For each time series, the global minimum and global maximum values are calculated before the segmentation, and the range is calculated as their difference. Next, the range is divided by the number of intervals *h*, thus facilitating calculation of the bounds of the equal-width intervals. The number of equal-width intervals *h* is calculated as *h* = ⌈*log*_2_ *n* + 1⌉, based on the Sturges rule [62]. The number of intervals *h* and the bounds of each interval are calculated only once, before the segmentation and feature extraction. When a particular window of time series data is processed, the values $x_{i}^{j}$ are discretized using the calculated bounds of the intervals for *j*-th time series. Finally, a histogram with *h* values is created which reflects the number values $x_{i}^{j}$ that are in a particular interval. Thus, the histogram (counts of values in each interval) represents the *h* generated features with this approach.

#### Percentile based features

Studies [58, 59] showed that percentile-based features are useful for activity recognition. Given a vector *x*_*i*_ of length *n*, the *q*-th percentile of *x*_*i*_ is the value *q*/100 of the way from the mimumum to the maximum in a sorted copy of *x*_*i*_ [63]. The percentile is the same as the minimum if *q* = 0 and the same as the maximum if *q* = 100. We have calculated the following percentile-based features from the values *x*_*i*_ of a time series in a particular sliding window segment: first quartile (*Q*_1_ obtained for *q* = 25), median (obtained for *q* = 50), third quartile (*Q*_3_ obtained for *q* = 75), inter-quartile range (*IQR* = *Q*_3_ − *Q*_1_) and 10 additional percentiles obtained for *q* ∈ {5, 10, 20, 30, 40, 60, 70, 80, 90, 95}. Thus, from one time series, 14 features are generated.

#### Correlations

The correlation between pairs of raw time series values within one window is used as informative features [57, 60]. In this study we used the correlation between the magnitude of the hip and ankle accelerometers.

The most widely used measure of dependence between two series, *x*_*i*_ and *y*_*i*_, is the Pearson’s correlation coefficient, as defined with Eq (1), where *n* is the number of values in *x*_*i*_ and *y*_*i*_, and in all sums 0 ≤ *i* < *n* stands:

$$\begin{array}{c} \frac{n\sum x_{i}y_{i}-\sum x_{i}\sum y_{i}}{\sqrt{n\sum x_{i}^{2}-{\left(\sum x_{i}\right)}^{2}}\sqrt{n\sum y_{i}^{2}-{\left(\sum y_{i}\right)}^{2}}} \end{array}$$ (1)

#### Autocorrelation

Auto-correlation of the measurements within one window can provide informative features too [57, 60]. Autocorrelation, also known as serial correlation, is the correlation of a signal with a delayed copy of itself as a function of delay. In other words, it is the similarity between observations as a function of the time lag between them. The analysis of autocorrelation can help in discovering repeating patterns, such as the presence of a periodic signal obscured by noise. It is often used in signal processing for analyzing functions or series of values, such as time domain signals. Let *τ* denote the lag. We define its domain with Eq (2):

$$\begin{array}{c} \tau \in \left\{\left\lfloor n/2\right\rfloor \right\}\cup \left\{2^{i}\;|\;1\leq i\leq log_{2}n\;\wedge \;2^{i}\leq n/2\right\} \end{array}$$ (2)

When *τ* = ⌊*n*/2⌋, the autocorrelation of the first half and second half of the signal will be computed, which can give some insights whether the signal is periodical. The other exponentially increasing values of the lag *τ* are heuristically determined and enable computation of the autocorrelation for a reasonable number of different lags. It is a balance between computing a lot of, potentially redundant, correlation coefficients (e.g. if *τ* increases linearly) and computing only several coefficients for predetermined values of *τ* (which do not take into account the length of the sliding window).

The classical autocorrelation is defined as:

$$\begin{array}{c} \frac{1}{\left(n-\tau \right)\sigma^{2}}\sum_{i=0}^{n-\tau -1}\left(x_{i}-\bar{x}\right)\left(x_{i+\tau }-\bar{x}\right) \end{array}$$ (3)

In addition to the classical autocorrelation, for the same values of *τ* as described earlier, the Pearson correlation is calculated as well.

In this study, per Eq (2) for the 60s windows *τ* ∈ {1, 2, 4, 30} and for the 180s windows *τ* ∈ {1, 2, 4, 90}. Consequently, for both windowing configurations there are four *τ* values which are used to calculate the classical autocorrelation and the Pearson correlation, yielding 8 features per time series in total.

#### Curve fitting parameters

The linear curve fitting parameters (2 coefficients needed to define the most optimal line that fits the *x*_*i*_ values) and the quadratic curve fitting parameters (3 coefficients needed to define the most optimal parabola that fits the *x*_*i*_ values) have been used as informative features in [57]. By performing linear and quadratic interpolation, 5 features are generated from each time series.

### Feature extraction and selection

In this subsection we describe the applied process of feature extraction, feature selection and classification.

Due to the data diversity with respect to the variety of field conditions under which it was collected, the key tasks are feature extraction from the raw accelerometer measurements and selection of the best feature set. A challenge for building robust features is dealing with drift in the data, which can be the result of: data generated by different sensors, data collected from different participants, loss of accuracy of accelerometers over time, etc. Ideally, such variations should have little to no effect on the trained classification models.

Depending on the problem domain, the types of extracted features are usually features that have been previously successfully applied to the same or a similar domain. This is often subjective and depends on the researcher’s experience. To alleviate this, we have systematically engineered new time series derived from the 12 original time series from both accelerometers and extracted a variety of features from all of them.

**Fig 3:**
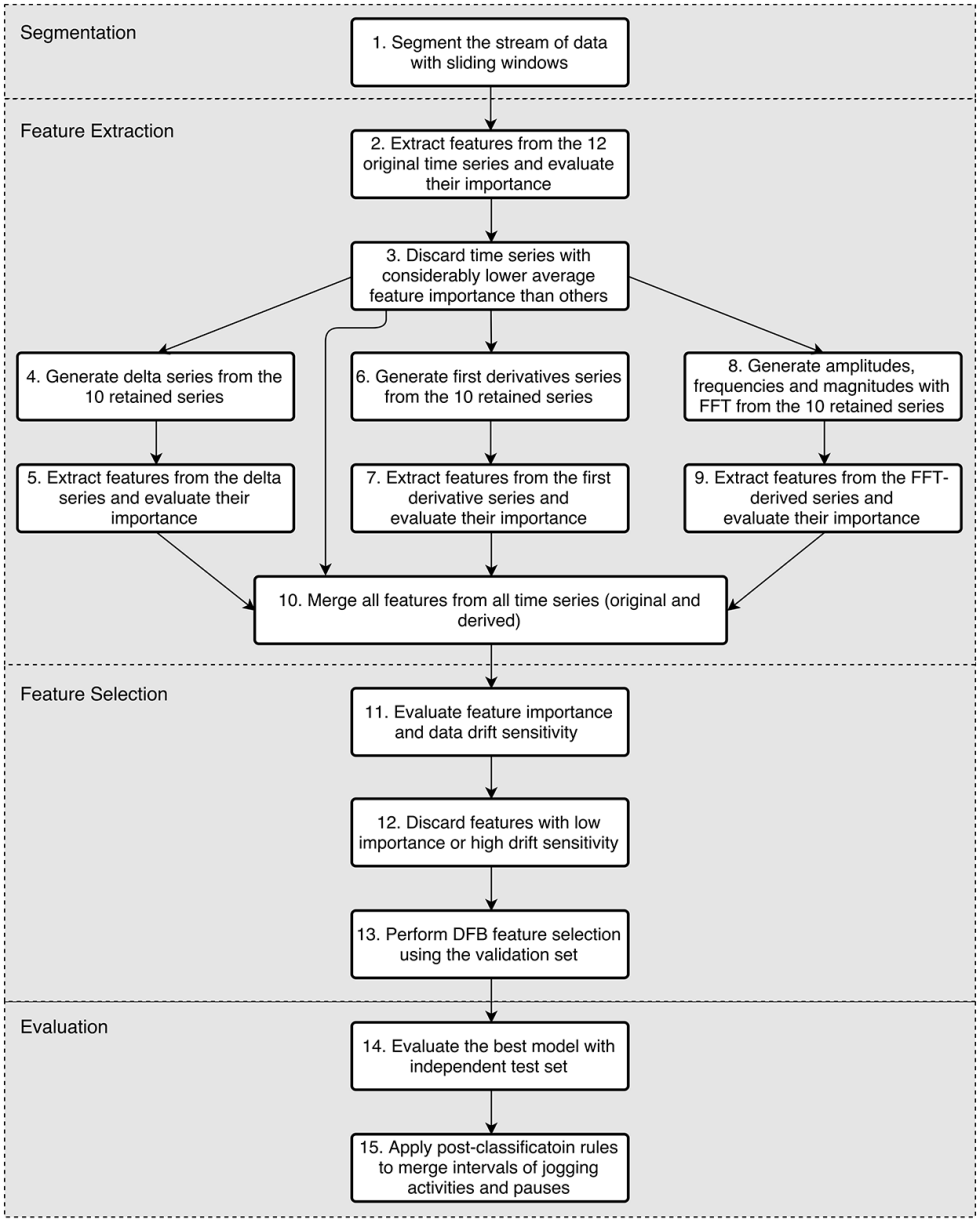
Algorithm for feature extraction, selection and classification.

The process flow, containing feature extraction, feature selection and classification steps, is shown in Fig 3.

In *step 1*, the data stream was segmented with the sliding windows technique, as described previously (see Table 2).

In *step 2* the nominal attribute (participants’ gender) was processed, i.e. it was transformed into two binary indicator values. A third numeric value was also generated from the gender using the Weight of Evidence (WoE) technique [64]. Additionally, in step 2 features from the windows of all 12 original time series were extracted and their individual importance was evaluated. From each time series, the following features were generated: 14 basic statistics, 7 histogram based features for the 60s windows (or 9 for the 180s windows), 14 percentile based features, 8 auto-correlation based features, and 5 curve fitting features. Therefore, there were 14 + 7 + 14 + 8 + 5 = 48 features per time series (50 for the 180s windows), plus 1 inter-correlation feature and 3 features based on the gender, yielding 12 × 48 + 1 + 3 = 580 features in total (604 for the 180s windows).

In *step 3*, the average feature importance per time series was computed, by training an ERT classifier and using its feature importance estimates. If the average importance of a time series was considerably lower than the most informative time series (i.e. less than 3 times), it was discarded, along with its features. In this study, the inclinometer time series from the ankle and hip were on average over 5 times less informative than the most informative time series. Therefore, these 2 time series were discarded, leaving the number of remaining features 580 − 2 × 48 = 484 (504 for the 180s windows).

During *step 4*, delta series were generated as follows. First, the mean value ${\bar{x}}_{i}$ of the measurements $x_{i}^{j}$ within a window of the i-th time series was calculated (0 ≤ *j* < *n*, where *n* is the number of measurements within one window). Then, the differences $\Delta x_{i}^{j}={\bar{x}}_{i}-x_{i}^{j}$ between the original measurement $x_{i}^{j}$ and the mean ${\bar{x}}_{i}$ was calculated. Note that the mean was calculated separately for each window and each time series. Thus, each original measurement $x_{i}^{j}$ was mapped to a new value $\Delta x_{i}^{j}$. As a result, this step generated 10 delta series from the 10 retained time series in this study.

In *step 5*, only 7 histogram based features (for the 60s windows or 9 for the 180s windows) and 14 percentile based features were calculated from the delta series. The auto-correlations features were omitted because they would be redundant to the auto-correlation features extracted from the original time series. Since the delta series is only a linear translation of the original series, the curve-fitting features and basic statistics features would be also redundant. This step from the 10 delta time series generated 10 × (7 + 14) = 210 features for the 60s windows (10 × (9 + 14) = 230 for the 180s windows).

In *step 6*, 10 first derivatives time series were generated from the 10 original time series. The first derivative is defined as difference between consecutive measurements within one window. Thus, from an original time series with *n* measurements within one window, the first derivative time series has *n* − 1 values.

In *step 7* features were extracted from the 10 first derivative time series in an analogous manner as from the original time series. The only difference is that auto-correlation features were not computed for the reasons described in step 5. As a result, this step generated 10 × (14 + 7 + 14 + 5) = 400 features in total for the 60s windows (10 × (14 + 9 + 14 + 5) = 420 for the 180s windows).

In *step 8* the Fast Fourier Transformation (FFT) of the 10 original time series was computed, transforming the accelerometry signals into frequency domain. As a result, 3 FFT-derived series of values from each original time series were generated: the series of frequencies, the series of amplitudes and the series of magnitudes, i.e. 30 FFT-derivied time series in total.

In *step 9*, for each of the 30 FFT-derived time series, the following features were extracted: minimum, maximum, mean, standard deviation, range, first and third quartile, inter-quartile range, median and the 10-th, 40-th, 60-th and 90-th percentile. The rationale was that these 13 features would sufficiently describe the distribution of values of the FFT-derived time series. The spectral centroid was feature computed by the FFT from each of the 10 original time series, therefore there were genereted 10 such features, or 30 × 13 + 10 = 400 frequency domain features in total.

In *step 10* all features generated by steps 2, 5, 7 and 9 were merged, resulting in one feature set containing 1494 features for the 60s windows: 484 from the 10 retained original time series, 210 from the 10 delta series, 400 from the 10 first derivative series, and 400 from the 30 FFT-derived series. For the 180s windows from each time series 2 additional histogram-based features are generated, thus in total there were 1554 features.

In *Step 11* the *feature importance* in the merged feature set was calculated by training an ERT classifier. With the method proposed in [65] the concept distribution drift of the features was estimated, by generating an artificial dataset containing all rows of the training and validation datasets. An artificial target label (i.e. class) was generated, which denoted from which dataset the corresponding row originates. On this artificial dataset an ERT classifier was trained and the importance of each feature was evaluated. The latter feature importance in fact defined the *data concept drift sensitivity* estimate of the feature, i.e. the very informative features for the artificial dataset were very sensitive to data distribution drift and therefore could potentially lead to model overfitting [65].

In *step 12*, from the feature importances and concept drift sensitivity of all features, the following 9 percentiles were calculated: 10, 20, 30, 40, 50, 60, 70, 80, 90. There were 9 percentiles from the feature importances and 9 percentiles from the concept drift sensitivity estimates. These 2 sets of percentiles were calculated and used as thresholds for feature importance and concept drift sensitivity. It evaluated all 9 × 9 = 81 combinations of thresholds, aiming to discard features which had low feature importance or high concept drift sensitivity. For each of the 81 feature sets, it build logistic regression models using the training dataset and evaluated them with the validation dataset. In this study, the number of retained features after this step was about 400. The purpose of this step was to significantly reduce the feature set size by discarding features with low importance of high concept drift sensitivity.

In *step 13* diversified forward-backward (DFB) feature selection was performed using a modified version of the algorithm described in [66]. It used logistic regression as a wrapper algorithm. Firstly, our approach ranked by importance the 400 features retained after step 12. Next, in one iteration of the forward pass, all available features were considered for addition. Starting from an empty feature set, it subsequently added features to the current best feature set, evaluated multiple feature sets in parallel and retained the features whose addition improved the predictive accuracy. We applied heuristic: features which did not improve the score when added to some feature set, will not be considered for addition to other feature sets tested later. The forward pass ended after all eligible features for addition were considered and there was no improvement of the best accuracy. Next the backward phase followed, which tested if the removal of any feature from the best feature set improved the score. In case a removal of a feature improved the accuracy, it started a new backward iteration. Otherwise, when all features were tested for removal the backward iteration stopped. In case of an improvement during the forward or the backward phase, the algorithm started a new cycle of forward and backward passes. Otherwise, the search converged and terminated. The algorithm also terminated when the maximum number of allowed feature sets were reached. The maximum was set to 2000 in this study, which was sufficient considering the maximum feature set size of 1494 features. The feature screening performed in step 12 was particularly important for this step because without it the search space would be significantly more complex.

We acknowledge that the selected best feature set might have been biased towards logistic regression, because it was used as a wrapper classification algorithm in step 13. As step 13 adds and removes features one-at-a-time, the wrapper algorithm needs to be unambiguously sensitive to such changes. Due to the randomness in the learning process, ERT and RF are not suitable for this step. Similarly, SVMs have limited applicability for step 13 because of their high sensitivity to parameter tuning. If they were used, then for each feature set parameter tuning needs to be performed, thus significantly increasing the required number of evaluations. Learning SVM models is generally significantly slower than logistic regression models (e.g. see Tables 3 and 4), so this would further slow down the feature selection by an order of magnitude. Naïve Bayes classifiers would be also applicable for step 13, as they are both fast and also their performance is impacted by additions and removals of features. However, prior applying them, the appropriate probability function (e.g. Gaussian, Bernoulli, etc.) needs to be determined, which would additionally complicate step 13. In [23] it was demonstrated that using Naïve Bayes and logistic regression for evaluating the impact of step-wise additions and removals of features during wrapper feature selection results in similar feature sets. For these reasons and owing to the simplicity and speed of the logistic regression algorithm, it was chosen as a wrapper algorithm for step 13. This allowed evaluating multiple feature sets in parallel, which is very efficient time-wise. Namely, the algorithm converged or evaluated a maximum of 2000 feature sets in less than 5 minutes when executing 12 threads in parallel. At the end of this step, the feature set that resulted in the highest accuracy for logistic regression was recorded and marked as the ‘best’ feature set.

**Table 3:** Performance of different classifiers on the 4 final feature sets, depending on feature type with the segmentation strategy of 60s windows without overlap.

Features	Classifier	Acc.	AUC	Prec.	Recall	Spec.	F1	Time
Best Ankle (8 feat.)	ERT	0.9970	0.9906	0.9339	0.8937	0.9988	0.9133	4.0
	RF	0.9969	0.9883	0.9579	0.8633	0.9993	0.9081	4.7
	Logistic	0.9967	0.9986	0.9259	0.8861	0.9987	0.9056	0.1
	SVM	0.9917	0.9940	0.7137	0.8962	0.9934	0.7946	24.3
Best Hip (20 feat.)	ERT	0.9967	0.9951	0.9171	0.8962	0.9985	0.9065	5.5
	RF	0.9962	0.9906	0.8939	0.8962	0.9981	0.8951	6.9
	Logistic	0.9977	0.9991	0.9528	0.9190	0.9992	0.9356	0.4
	SVM	0.9970	0.9988	0.9533	0.8785	0.9992	0.9144	188.2
All (17 feat.)	ERT	0.9954	0.9977	0.8600	0.8861	0.9974	0.8728	9.9
	RF	0.9959	0.9959	0.8961	0.8734	0.9981	0.8846	8.2
	Logistic	0.9963	0.9911	0.9023	0.8886	0.9982	0.8954	0.9
	SVM	0.9954	0.9987	0.8391	0.9241	0.9968	0.8795	249.9
Best Ankle + Best Hip(28 feat.)	ERT	0.9971	0.9966	0.9321	0.9038	0.9988	0.9177	7.8
	RF	0.9972	0.9911	0.9514	0.8911	0.9992	0.9203	7.7
	Logistic	0.9968	0.9992	0.9030	0.9190	0.9982	0.9109	0.5
	SVM	0.9976	0.9993	0.9525	0.9139	0.9992	0.9328	204.7

Classifiers are ERT for Extremely Randomized Trees, RF for Random Forest, Logistic for logistic regression, SVM for Support Vector Machines. The SVM parameters for the first feature set are: *C* = 10, *γ* = 0.01 and for all remaining feature sets: *C* = 0.1, *γ* = 0.0001. Acc. stands for Accuracy, AUC for Area Under the receiver-operating characteristic Curve, Prec. for Precision, Recall (also known as sensitivity, hit rate, or true positive rate), Spec. for Specificity (also known as true negative rate), F1 for F1 score (harmonic mean of precision and recall) and Time for Total time (in seconds) for building a model on the training dataset and making predictions on the test dataset. The classifier with gray background has highest accuracy for the feature set, thus was selected as best for that feature set. Other cells with gray background represent the best classifier for the feature set in regards to the metric (column).

**Table 4:** Performance of different classifiers on the 4 final feature sets, depending on feature type with the segmentation strategy of 180s windows with 120s overlap.

Features	Classifier	Acc.	AUC	Prec.	Recall	Spec.	F1	Time
Best Ankle (17 feat.)	ERT	0.9990	0.9930	0.9571	0.9750	0.9993	0.9659	5.0
	RF	0.9988	0.9928	0.9509	0.9688	0.9993	0.9598	6.1
	Logistic	0.9989	0.9994	0.9837	0.9406	0.9998	0.9617	0.3
	SVM	0.9994	0.9972	1.0000	0.9625	1.0000	0.9809	25.2
Best Hip (20 feat.)	ERT	0.9981	0.9976	0.9761	0.8938	0.9997	0.9331	7.1
	RF	0.9978	0.9975	0.9564	0.8906	0.9994	0.9223	5.6
	Logistic	0.9967	0.9988	0.8739	0.9094	0.9980	0.8913	0.4
	SVM	0.9968	0.9994	0.8862	0.9000	0.9983	0.8930	141.4
All(12 feat.)	ERT	0.9954	0.9977	0.8600	0.8861	0.9974	0.8728	9.9
	RF	0.9975	0.9935	0.9102	0.9188	0.9987	0.9145	5.5
	Logistic	0.9990	0.9962	1.0000	0.9344	1.0000	0.9661	0.3
	SVM	0.9972	0.9964	0.8862	0.9250	0.9982	0.9052	24.8
Best Ankle + Best Hip(37 feat.)	ERT	0.9988	0.9982	0.9399	0.9781	0.9991	0.9587	7.6
	RF	0.9983	0.9964	0.9354	0.9500	0.9990	0.9426	7.6
	Logistic	0.9985	0.9997	0.9470	0.9500	0.9992	0.9485	1.6
	SVM	0.9984	0.9998	0.9613	0.9313	0.9994	0.9460	186.9

All naming conventions are the same as in Table 3. The SVM parameters for the first and third feature sets are: *C* = 10, *γ* = 0.001 and for the second and forth feature sets: *C* = 0.1, *γ* = 0.0001.

Ultimately, once the best feature set was determined, the evaluation of the automated method for identification of jogging periods was performed in two phases. The first phase refers to *step 14* in Fig 3. In this step, first the optimal values for the SVM parameters C and *γ* are estimated. In the second part of step 14, all classification algorithms (Logistic regression, ERT, RF and SVM with optimal parameters) were evaluated using the independent test dataset.

The second phase of evaluation of the method for identification of jogging periods was performed in *step 15*. Post-classification rules, described in more detail in the following section, were applied to further improve the accuracy of the analysis.

### Evaluating accelerometer location usefulness

In order to assess whether recordings of two accelerometers, i.e. monitoring of body movement at two different body positions is required or whether one position is sufficient to accurately detect jogging periods, we have applied the following approach. Step 13 on Fig 3, i.e. DFB feature selection, was executed three times so it considers features only from a particular sensor location:

1. **Ankle features**. DFB feature selection considered only features extracted from the accelerometer placed at the ankle and determined the best feature set. In essence, only features from the 6 original time series (reduced to 5 in step 3) from the ankle accelerometer and their derived series (steps 4 to 9) were used as input to this step.
2. **Hip features**. DFB feature selection considered only features extracted from the accelerometer placed at the hip and determined the best feature set. Similarly, only features from the 6 original time series (reduced to 5 in step 3) from the hip accelerometer and their derived series (steps 4 to 9) were used as input to this step.
3. **All features**. DFB feature selection considered all features extracted from both the hip and ankle accelerometers and determined the best feature set. Therefore all features from the 12 original time series (reduced to 10 in step 3) from both accelerometers and their derived series (steps 4 to 9) were used as input to this step.

Thereupon, steps 14 and 15 were executed three times to build classification models from the three feature sets obtained by the three executions of step 13. Additionally, steps 14 and 15 were executed a forth time using the union of the best feature sets per accelerometer location (i.e. union of items 1 and 2 in the previous list). The purpose of the fourth feature set (regarded as ‘**Best Ankle + Best Hip**’ henceforth), is supplementary to the third feature set. Generally, the third and fourth feature sets can be different because the DFB feature selection is not guaranteed to find globally optimal feature sets, considering that its search strategy is highly greedy.

### Post-classification rules

By analyzing the jogging periods based on the golden standard labels (see Table 1 in the Data management subsection), we were able to identify the distribution of the duration of jogging periods and the duration of pauses between consecutive jogging periods that are not too far apart, i.e. are smaller than an hour and a half. These distributions are shown in Fig 2. Aiming to improve the recognition of jogging periods with reasonable durations and pauses between them, we have defined the following rules, executed in the order they are defined:

**Rule 1** Remove jogging periods that are shorter than or equal to 3 minutes.
**Rule 2** Apply Rule 1 and afterwards merge adjacent jogging periods if the pause between them is shorter than or equal to 5 minutes.
**Rule 3** Apply Rule 2 and afterwards merge adjacent jogging periods if the pause between them is shorter than or equal to the sum of their durations.

After merging adjacent jogging periods because of Rule 2 and Rule 3, the pause between them is no longer considered as a pause, rather it is a part of the resulting jogging period whose total duration is the sum of the durations of the two merged jogging periods and the pause. The motivation for defining these rules are the field conditions under which the accelerometer data was collected. The rules try to account for typical jogging scenarios. The rationale for Rule 1 is that jogging periods shorter than or equal to 3 minutes are very unlikely for intended sport rather they typically occur if the participant had a short burst of running (e.g. to catch the bus). Such periods could be recognized by the classification algorithms, because the nature of the recorded accelerometer readings could fit the model for running. However, the health-associated benefits from such short and isolated periods of running are questionable [1, 2, 37, 40] and our intention was to detect the intended jogging periods really done for sport. The rationale for Rule 2 and Rule 3 is that a short pause of couple of minutes can be allowed because the participant could be: waiting for a traffic light for crossing a road, doing some stretching, have a little talk when meeting a friend, etc.

**Fig 4:**
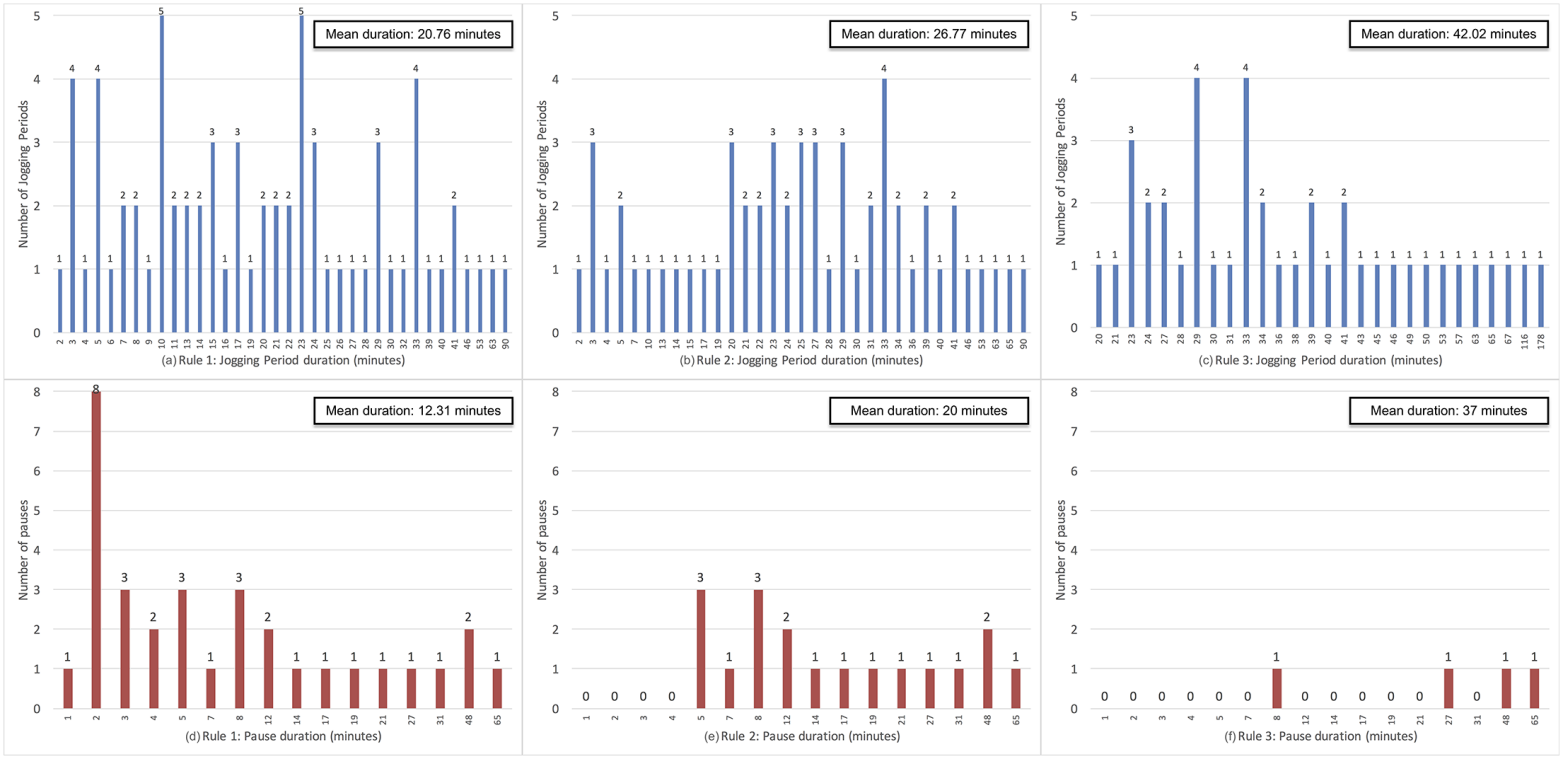
Distribution of the duration (in minutes) of jogging periods ((a), (b) and (c)) and pauses between jogging periods ((d), (e) and (f)) in the training and validation datasets based on the ‘golden standard’ labels after applying post-classification Rule 1 ((a) and (d)), Rule 2 ((b) and (e)) and Rule 3 ((c) and (f)).

After applying Rule 1, some of the shorter jogging periods in Fig 2(a) were removed, thus obtaining the distribution shown in Fig 4(a) and 4(d). Likewise, after applying Rule 2, some jogging periods were merged, including the pause between them. Consequently, some of the shorter pauses in Fig 2(b) were removed, thus obtaining the distribution shown in Fig 4(b) and 4(e). For instance, if there was a pause of 3 minutes between two jogging periods of 5 and 7 minutes, after applying Rule 2 they were merged, resulting in one 15 minute jogging period. Similarly, Rule 3, which is a more dynamic extension of Rule 2, merged jogging periods in case their total duration was greater than the pause between them. For example, jogging periods of 4 and 10 minutes separated by a pause of 6 minutes would be merged, resulting in one jogging period of 20 minutes. After applying Rule 3, the final distribution of jogging periods per duration and pauses between jogging periods per duration is shown in Fig 4(c) and 4(f). Note that data presented in Figs 2 and 4 derives from the golden standard labels for the participants in the training and validation datasets. The test dataset was intentionally left out of the analysis so it can remain independent and not influence the reasoning and definition of rules.

The purpose of the rules was to smooth and amend both the golden standard periods, as well as the ones that were automatically recognized with the machine learning algorithms. Therefore, the rules were applied on the golden standard periods and the recognized periods by the 4 classification algorithms (RF, ERT, logistic regression, SVM) using the best feature sets.

### Converting predictions to jogging periods

However, for the segmentation strategy of 180s windows with 120s overlap, a same time period of 60s is part of 3 consecutive windows that could potentially have different predicted activities. In that case, the final prediction for a 60s period is obtained by majority vote of the 3 possible predictions from the 3 windows in which the 60s belong to. Potentially, a 60s jogging period could be preceded and followed by non-jogging activities. Therefore, because of the majority vote, it could be outvoted and classified as non-jogging. This is physiologically plausible and corresponds to the realistic conditions in the field. However, as discussed in the previous section, such isolated jogging periods are not of interest and would be removed by Rule 1. Similarly, an isolated pause could be outvoted and classified as jogging. Again, this is plausible and relates to the purpose of Rule 2 and Rule 3.

### Jogging period matching ratio

Considering the segmentation strategy of 60s and 180s windows, the datasets exclude border periods where the activity changes from jogging to non-jogging or vice versa. Therefore, those cases are not reflected in common classifier performance metrics, such as accuracy. Moreover, when there is an overlap between adjacent windows, the accuracy is more difficult to interpret in terms of jogging periods. On the other hand, comparing the jogging periods of recognized activities addresses border periods, as well. To facilitate comparison of classification algorithms in terms of recognized jogging periods, we define a metric jogging period *matching ratio* with Eq (4). The length of correctly identified jogging period is *matched*, the length of jogging period not identified by the classification algorithm is *missed*, and the length of jogging period that were recognized, but were not present in the golden standard labels are *other*.

$$\begin{array}{c} matching\_ratio=\frac{matched}{matched+missed+other} \end{array}$$ (4)

The parameters used in Eq (4) intuitively correspond to the values in a classifier confusion matrix. More precisely, *matched* relates to the True Positives, *missed* to the False Negatives and *other* to the False Positives.

The jogging period matching ratio was computed after the best feature sets were determined for each sensor position. All classification models were also compared by it, to complement the more standard algorithm performance metrics.

## Results

In this section we report the performance of the best feature set when the training dataset is used for building classification models and the test set for evaluating the performance of the algorithms. For each segmentation strategy, we used features extracted only from the accelerometer placed at the hip, only from the accelerometer placed at the ankle or from both accelerometers. Ultimately, we applied post-classification rules aiming to further improve the performance.

### Classifier performance

Table 3 shows the performance of the 4 different classifiers with the 4 final feature sets, depending on feature types when using windows of 60s without overlap.

It is apparent that classifiers have very high accuracy (over 0.995). The logistic regression is by far the fastest algorithm for building classification models and making predictions, while offering best performance in almost all metrics and feature sets. Furthermore, the feature set based only on the hip accelerometer results in a similar performance as when using both accelerometers for all classification algorithms.

Table 4 shows the performance when using 180s windows. In this case all classifiers have very high accuracy (over 0.995). Regarding the accelerometer position, using the features only from the ankle accelerometer yielded better performance than the ones from the hip accelerometers, and was comparable to the feature sets from both accelerometers.

The feature importance estimated by Random Forest with 1000 trees with the Best Ankle + Best Hip feature set and the 180s windows are provided in Table 5. The best set was composed of features from all time series (original and derived), thus justifying the generation of new time series (steps from 4 to 9 in Fig 3). There were features from all types: basic statistics, percentiles, auto-correlation and histograms. Noteworthy, there are not so many FFT-based features in the final set. This is because these features are very sensitive to concept drift, which is a consequence of the difference in gait between the participants in the train and validation sets. In fact, when features were ranked by concept drift in descending order in step 12 in Figs 2 and 3 for the 180s windows, in the top 100, 200 and 300 features, there were 75, 128 and 180 FFT-based features, respectively. On the other hand, when the features were ranked by importance in descending order, in the top 100, 200 and 300 features, there were only 24, 38 and 54 FFT-based features, respectively. Therefore, most of the FFT-based features are discarded in step 12 in Fig 2.

**Table 5:** Feature importances estimated by Random Forest with 1000 trees with the Best Ankle + Best Hip feature set and the segmentation strategy of 180s windows with 120s overlap.

#	Feature name	Score	#	Feature name	Score
1	AnkleSteps auto-corr t = 1	0.1495	20	HipAxis1 quad fit c2	0.0045
2	AnkleSteps auto-corr t = 2	0.1239	21	FFT_amp(AnkleMag) IQR	0.0045
3	AnkleMag auto-corr t = 2	0.1065	22	1st_deriv(HipSteps) perc. 70	0.0044
4	AnkleSteps perc. 80	0.0980	23	HipAxis1 lin fit c1	0.0036
5	AnkleMag energy	0.0945	24	FFT_mag(AnkleSteps) perc. 90	0.0031
6	AnkleSteps hist [0, 0.57)	0.0725	25	FFT_freq(AnkleSteps) IQR	0.0031
7	HipAxis2 median	0.0716	26	FFT_freq(AnkleMag) max	0.0030
8	HipAxis3 perc. 60	0.0474	27	1st_deriv(AnkleMag) hist [-0.5, 0.5)	0.0029
9	HipAxis2 perc. 10	0.0332	28	delta(AnkleSteps) perc. 80	0.0028
10	HipAxis2 perc. 5	0.0324	29	delta(AnkleMag) perc. 80	0.0024
11	HipAxis2 perc. 70	0.0297	30	HipSteps perc. 80	0.0023
12	HipAxis1 perc. 80	0.0291	31	1st_deriv(HipSteps) perc. 20	0.0017
13	HipAxis1 perc. 10	0.0174	32	HipAxis1 hist [129, 259)	0.0014
14	HipMag median	0.0106	33	AnkleAxis2 min	0.0010
15	AnkleMag perc. 10	0.0101	34	HipAxis3 hist [0, 129)	0.0010
16	HipAxis2 min	0.0101	35	HipAxis2 hist [0, 129)	0.0008
17	HipAxis2 quad fit c2	0.0098	36	1st_deriv(AnkleSteps) hist [-2.9, -1.7)	0.0006
18	HipAxis1 min	0.0052	37	delta(HipAxis3) hist [-70, 89)	0.0004
19	delta(AnkleMag) perc. 75	0.0050			

### Comparison to a baseline model

Aiming to compare our feature engineering approach with a baseline feature set, we have applied the feature extraction method described in [67] and used in [26]. The first five coefficients of the FFT power spectrum for each of the 10 retained time series were used as baseline feature set, as they yielded the best performance for a similar sensor setup (thigh and ankle accelerometers) [67]. Thus, a set of 10 × 5 = 50 features was generated from both accelerometers, and two additional feature sets of 25 each were generated from either the ankle or hip accelerometer. It was done for both segmentation strategies. Using these features, we trained the same 4 classifiers (i.e. RF, ERT, Logistic regression and SVM including parameter tuning). To summarize the results, we have computed the maximum accuracy, AUC, precision, recall and F1 score per sensor location from both approaches (i.e. baseline and proposed), as shown in Table 6. It is evident that for almost all metrics and sensor locations, most notably for the precision, recall and F1 score, the proposed method provides better results. The effect is more evident in terms of matching ratio, as described in the following subsection.

**Table 6:** Comparison of the baseline and proposed approach for feature extraction per sensor location by the best obtained value per metric.

	Method	Accuracy	AUC	Precision	Recall	Specificity	F1
Ankle	Baseline	0.9982	0.9993	0.9557	0.9749	0.9993	0.9393
	Proposed	0.9994	0.9994	1.0000	0.9750	1.0000	0.9809
Hip	Baseline	0.9977	0.9993	0.9426	0.9469	0.9991	0.9187
	Proposed	0.9981	0.9994	0.9761	0.9190	0.9997	0.9356
Ankle + Hip	Baseline	0.9987	0.9997	0.9579	0.9563	0.9993	0.9563
	Proposed	0.9990	0.9998	1.0000	0.9781	1.0000	0.9661

All naming conventions are the same as in Table 3.

### Evaluation on balanced datasets

Due to the high class imbalance in the datasets (see Table 2), there is a possibility for over-fitting. To investigate whether that is the case, we have balanced the datasets by stratified under-sampling of the majority class. After the random under-sampling of the training, validation and test sets, for each participant there was an equal number of jogging and non-jogging instances. The non-jogging instances were randomly selected from all non-jogging instances of the participant.

Next, for both segmentation strategies using the 4 classifiers (i.e. RF, ERT, Logistic regression and SVM) different models were built using the same feature sets that were identified by the proposed algorithm for each sensor location and additionally, using the baseline features described in the previous subsection. Note that the feature sets used for the balanced datasets are the ones identified with the feature selection process applied on the original datasets. For the balanced datasets, the models were built from the training set and evaluated on the test, while SVM parameter tuning was performed using the validation set.

The whole process of random under-sampling was repeated 100 times for each segmentation strategy and Table 7 shows the average performance on the balanced datasets of the baseline and proposed approach for feature extraction per sensor location by the best obtained result per metric. Evidently, for almost all metrics and sensor locations, the proposed method provided better results, especially when both sensors are used. This demonstrates that the selected feature sets by the proposed method on the highly imbalanced datasets are applicable even on balanced datasets and are again better than the baseline method.

**Table 7:** Average performance on the balanced datasets of the baseline and proposed approach for feature extraction per sensor location by the best obtained value per metric.

	Method	Accuracy	AUC	Precision	Recall	Specificity	F1
Ankle	Baseline	0.9825	0.9947	0.9788	0.9851	0.9786	0.9820
	Proposed	0.9827	0.9980	0.9789	0.9865	0.9787	0.9827
Hip	Baseline	0.9830	0.9969	0.9803	0.9859	0.9789	0.9830
	Proposed	0.9847	0.9971	0.9792	0.9904	0.9801	0.9848
Ankle + Hip	Baseline	0.9817	0.9958	0.9780	0.9857	0.9777	0.9818
	Proposed	0.9841	0.9972	0.9787	0.9899	0.9784	0.9842

All naming conventions are the same as in Table 3.

### Post-classification rules and jogging period matching ratio

The jogging period matching ratio considers border periods where the activity changes from jogging to non-jogging or vice versa. These cases are not reflected in the classification accuracy and other metrics provided in Tables 3, 4 and 6, especially when the adjacent windows overlap.

**Fig 5:**
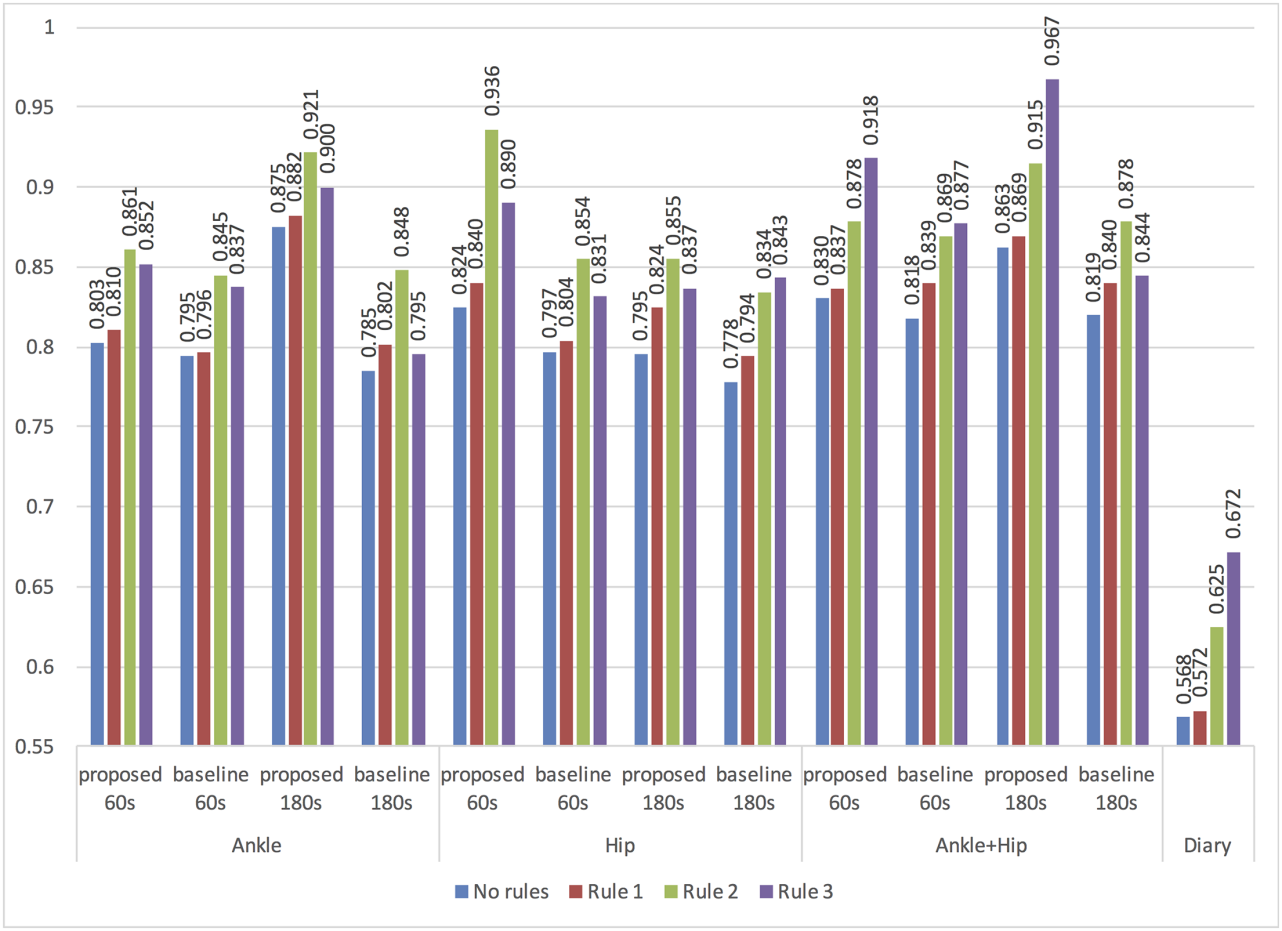
The jogging period matching ratio per feature set type and applied post-classification rule for the highest-accuracy classification model obtained with the proposed and baseline feature sets.

The post-classification rules were applied to the predictions of the classification model that resulted in best accuracy per accelerometer position and segmentation strategy, and the jogging period matching ratio was calculated, as shown in Fig 5. The feature sets obtained with the proposed method result in better matching ratio compared to the baseline feature sets regardless of the accelerometer position and segmentation strategy. This still stands after applying the post-classification rules. For all feature sets, accelerometer position and segmentation strategy, Rule 1 and Rule 2 improve the jogging period matching ratio, whereas Rule 3 only sometimes improves it. All rules improve the matching ratio for the diary labels.

## Discussion

This study introduces a new algorithm based on machine learning techniques for detection of intended jogging periods, using conventional accelerometer data recorded under field conditions. Different classifiers with different feature selection methods have been applied using only data from the accelerometer placed at the hip, from the accelerometer placed at the ankle, or from both accelerometers. We found that for different evaluation metrics, different classification models outperform depending on the chosen feature set (Tables 3 and 4). Our approach used two different window lengths for segmentation. For the first segmentation strategy (i.e. 60s windows without overlap), the best accuracy is obtained using the accelerometer placed at the hip, while for the second strategy (i.e. 180s windows with 120s overlap), the accelerometer placed at the ankle performs better. However, both strategies result in a very good accuracy of at least 0.99 and are superior to the baseline approach for feature extraction (see Table 6) in regards to multiple metrics. Further, for both segmentation strategies it became evident that using all features from both sensors does not offer a significant improvement than using only one sensor.

To better estimate the performance of the models, we calculated the jogging period matching ratio, using the classification models that had best accuracy for a particular feature set and per segmentation strategy (best classifiers are marked with gray background in Tables 3 and 4). The highest matching ratio achieved by the proposed method was 0.875 and was obtained with the 180s windows and only the ankle accelerometer, whereas the best baseline result was 0.819 with both accelerometers (Fig 5). In fact, the best proposed models yielded significantly better matching ratio than the best baseline models regardless of the accelerometer position or window length. In respect to the segmentation strategy, the 60s and 180s windows had best matching ratio of 0.83 and 0.875, respectively.

To embed different jogging scenarios under field conditions, we applied 3 post-classification rules. After applying Rule 1, which removes jogging periods that are shorter than or equal to 3 minutes, the matching ratio was improved regardless of the feature set (proposed or baseline), segmentation strategy or accelerometer position (see Fig 5). The best matching ratios were 0.882 and 0.84 for the proposed and for the baseline method, respectively. The 60s and 180s windows resulted in 0.84 best matching ratio of 0.830 and 0.882, respectively.

Likewise, after applying Rule 2, which merges adjacent jogging periods if the pause between them is shorter than or equal to 5 minutes, matching ratio was improved in all cases even more dramatically than after applying Rule 1. The 60s windows resulted in best matching ratio (0.936) compared to the matching ratio of the 180s windows (0.921, see Fig 5). Generally, Rules 1 and 2 increased the matching ratio for all algorithms, segmentation strategies and feature sets, while being very intuitive and clear. Another notable consequence of Rule 2 is that the best matching ratio for both segmentation strategies results from the data of one of the accelerometers (i.e. the hip accelerometer for the 60s windows, and the ankle accelerometer for the 180s window). Therefore, there is no benefit from using both sensors in terms of obtaining the best matching ratio after applying Rule 2. Using two accelerometers doubles the number of available features compared to when only one accelerometer is used, thus making it more difficult for the feature selection algorithm to find the optimal feature set. The best matching ratio of the baseline approaches was 0.878, which is significantly less than the matching ratio 0.936 obtained the best proposed model for the hip accelerometer and 60s windows.

Rule 3, which merges adjacent jogging periods, is slightly more complex than Rule 1 and Rule 2. Even though the overall best matching ratio (0.967) was obtained (Fig 5) after applying Rule 3, in most cases Rule 3 actually degraded the matching ratio, especially for the longer windows. This is also the case for baseline models. A classification error (i.e. incorrectly recognizing a jogging activity when the participant is not jogging or vice versa) at the beginning and the end of the window degrades the matching ratio more for longer windows than for shorter ones. For example, Rule 3 will merge a jogging period of 15 minutes, followed by a non-jogging period of 30 minutes and then by a jogging period of 20 minutes into one 65 minute jogging period. Such cases are plausible (e.g. jogging periods separated by another type of physical exercise), but Rule 3 would misinterpret them as one jogging period. On the contrary, Rule 2 limits the duration of the pause to maximum 5 minutes to reduce/minimize the impact of misclassifications. Because Rule 3 is dynamic, in some corner-cases it might even exacerbate the performance, therefore we suggest that Rules 1 and 2 are sufficient for this domain.

Fig 5 shows that Rules 1, 2 and 3 improved the diary matching ratio from 0.568 to 0.572, 0.625 and 0.672, respectively. Nonetheless, it was still significantly worse than all classification algorithms, regardless of the feature set or segmentation strategy. This may be related to the fact that reported jogging periods are subject to some uncertainties, such as accuracy of time and recollection of the actual time window. Consequently, we can conclude that keeping a diary for jogging periods activities has limited applicability because those periods can be detected more accurately using a machine learning approach. Another benefit of using automated activity recognition is reducing the intrusiveness to participants, which may lead to better consent to participate in the study.

Regarding the classifiers’ performance, SVMs required significantly more time than the other classifiers (Tables 3 and 4). Logistic regression was by far the fastest, and ERT and RF had comparable speed for this feature set size. SVMs had an additional complexity because their parameters’ tuning time is not included in the reported times. For the other classifiers, there was no benefit from parameter tuning. Regarding predictive performance, logistic regression is most consistent and often the best. However, this may be because it was the wrapper algorithm used during feature selection, hence the selected feature sets were most suited for it. Compared to a baseline method with hand-tailored features, the proposed approach for feature engineering resulted in overall better performance per accelerometer location across various metrics (see Table 6).

To investigate whether the performance would be improved with a larger training set, after finding the best feature sets per sensor location and segmentation window we have also repeated the experiments but used the union of the training and validation dataset (27 participants in total) for building a final classification models and the test dataset (12 participants) for evaluating their performance. The same process was repeated with the baseline feature sets, as well. The classification accuracy of different models varied insignificantly (changes usually only in the 4-th decimal of the statistics presented in Tables 3, 4 and 6), not enough to improve the jogging matching ratio significantly. The relative advantages and disadvantages of various feature sets, sensor locations, classification algorithms and segmentation windows were the same as when using only the set of 14 participants for training.

Based on the presented results, for detection of jogging periods we recommend: (i) using shorter windows because they could be used for detection of other activities with shorter duration; (ii) application of post-classification rules to boost the performance; (iii) using logistic regression because of its simplicity and speed, which allows real-time activity detection even for devices with limited resources (battery or computing power) like smartphones or wearables. However, if more activities need to be classified, similar study is needed to determine the most suitable parameters, classification algorithms, window size, sensor placement, domain-specific post classification rules, etc.

Other studies from the literature use accelerometer data to develop methods for specific movements detection, including jogging or walking.

The study in [27] identifies thirteen human activities using a dataset from 10 male participants generated in a controlled environment. They use three motion sensors (accelerometer, gyroscope and linear acceleration sensor) at both wrist and pocket positions. The combination of the two positions outperforms the wrist position alone, mainly at smaller segmentation windows, especially for less-repetitive activities. Similar to our findings, they also report very high jogging activity accuracy, using three classification algorithms: Naïve Bayes, k-Nearest Neighbors (kNN) and Classification Trees. The main advantage of our study is that our dataset is collected under field conditions with more participants (18 male and 21 female). Moreover, we are focused on recognizing intended longer jogging periods, whereas in [27] each activity was performed for only 3 minutes in total. Another advantage of our study is that all features in our final sets are systematically extracted and selected in an automated process, whereas in [27] the features have been manually generated based on literature study and by manual analysis of the raw data.

The experiments in [29] demonstrate an algorithm which determines the optimal number of activity sensors for accurate steps detection during dynamic activities in laboratory setting. They found that only the thigh-ankle combination or single waist sensor could accurately distinguish between walking and jogging steps. Even though their experimental setup differs from ours, our conclusions are coherent: the jogging activities can be accurately detected by one sensor at either position, hip or ankle.

A study presented in [25] detects single steps and falls using a single tri-axial accelerometer. The underlying idea was to implement an algorithm demanding minimal computational requirements directly in the sensor device to pre-process the sensed data stream before sending the information to a central point, where the information from different sensors is combined to improve the accuracy. Their approach relies on hand-tailored features, specific to the two atomic activities of interest. Although our study has generally different goals, the automated feature extraction and selection process can be used for identifying small number of features that are computationally efficient and can be also computed on hardware with limited resources. This can be a significant advantage over manually engineered features aimed at detection of different activities.

The study presented in [24] demonstrates how the same types of sensors as the ones used in our study can facilitate detection of driving periods. The approach is based on short-time Fourier transform applied to the raw accelerometry data and focuses on frequency vibration ranges that are specific to car driving. Although our results are not directly comparable, the high AUC of 0.94 presented in their study is encouraging for our future research, aimed at detection of other activities besides jogging.

The main discoveries of the study in [26] is that recalibrating the algorithm with data closer to real-life conditions from an independent group of subjects is useful. Albeit their goal was detection of sedentary behaviors, their findings highlight the main strengths of our study design: using data collected under field conditions and evaluating the algorithms on an independent group of participants.

Compared to other studies for activity recognition [27, 30, 32, 33], the final feature sets obtained by the proposed feature engineering method were considerably smaller (up to 20 features). This is important because it improves the speed of the classification models, while simplifying them. In comparison to studies that use small number of features [60], the advantage of our approach is the automatic identification of optimal feature sets, instead of using apriori determined features. Even though the proposed feature engineering methodology considers a high dimensional feature space, it eventually yields very concise and robust feature sets. As a matter of fact, it should be used only in the modeling phase of machine learning problems involving sensory data. Then, the outcome of the analysis (i.e. the final feature sets and the identified optimal machine learning algorithm) can be used for building and deploying in production low dimensional models.

## Conclusions

In this study, we have created a system for automatic identification of jogging periods of adolescents based on conventional accelerometer data collected under field conditions. Compared to a golden standard generated by visual inspection of data, the detected jogging periods by machine learning based approach were significantly more accurate than the reported jogging periods in participants diaries.

In order to improve the jogging matching ratio, i.e. the length of correctly identified jogging periods related to the total time including the missed one, we applying post-classification rules created by domain experts, which considered jogging breaks and very short jogging periods. We discovered that the post classification rules had significantly greater impact on improving the matching ratio than the choice of classification algorithm or sliding window length.

We have also analyzed the accuracy of jogging period identification depending on the number of accelerometers and their position (i.e. at the hip or ankle). We could show that by using the data from one accelerometer at either position (e.g. the commonly used hip) the jogging periods can be recognized with the same accuracy as when using data from two sensor locations, i.e. from hip and ankle. This can reduce the cost of epidemiological studies, as well as the intrusiveness to participants, because they would have to wear only one sensor instead of two. Hopefully, this may also increase the compliance of potential subjects to participate in the study. Since in epidemiological studies physical activity is typically monitored by an accelerometer placed at the hip, our algorithm can be applied to evaluate jogging periods retroactively.

Also, the findings of our study should be assessed for recognizing activities in independent cohorts of adolescents. This is important because other epidemiological studies have available data, but lack labels (even self-reported) and therefore have limited ability for estimation of physical activities. Another way to obtain labeled data is from a controlled environment, which could be utilized to train models, and use it for recognizing activities performed under field conditions.
